# Post-Chemotherapy Antibody-Based Continuation and Maintenance Strategies in HER2-Positive Metastatic Breast Cancer: A Translational Narrative Review

**DOI:** 10.3390/antib15020036

**Published:** 2026-04-16

**Authors:** Katarzyna Pogoda, Karolina Lewińska, Paulina Kalman, Anna Bałata, Piotr J. Wysocki

**Affiliations:** 1 Department of Breast Cancer and Reconstructive Surgery, Maria Sklodowska-Curie National Research Institute of Oncology, 02781 Warsaw, Poland; 2 Maria Sklodowska-Curie National Research Institute of Oncology, 02781 Warsaw, Poland; 3 Department of Oncology, Jagiellonian University-Medical College, 31-501 Krakow, Poland; piotr.wysocki@uj.edu.pl

**Keywords:** HER2-positive breast cancer, monoclonal antibody, maintenance therapy, CDK4/6 inhibitor, HER2 tyrosine kinase inhibitor, dual HER2 blockade, chemotherapy de-escalation, tumor biology, translational oncology

## Abstract

The treatment paradigm for HER2-positive metastatic breast cancer has evolved from continuous chemotherapy-based regimens to a model of finite chemotherapy induction followed by sustained antibody-driven disease control. The CLEOPATRA trial established dual HER2 blockade with trastuzumab and pertuzumab plus a taxane as the biological and clinical anchor of this approach, demonstrating that chemotherapy is administered for a defined induction period, after which antibody maintains disease suppression. An increasing body of clinical evidence indicates that antibody-based regimens can be combined with targeted agents, including CDK4/6 inhibitors or HER2 tyrosine kinase inhibitors, to achieve durable disease control without the need for continuous chemotherapy. In the PATINA trial, the addition of palbociclib to trastuzumab, pertuzumab, and endocrine therapy was associated with a clinically meaningful improvement in progression-free survival in hormone receptor-positive, HER2-positive metastatic breast cancer. At the same time, quality of life was maintained despite higher rates of hematologic toxicity. More recently, HER2-CLIMB-05 demonstrated that the addition of tucatinib to dual HER2 antibody therapy significantly prolonged progression-free survival, supporting a model of sustained, multi-agent HER2 pathway suppression. The monarcHER trial provided biological proof of concept that antibody plus CDK4/6 inhibition can achieve disease control without chemotherapy in hormone receptor-positive, HER2-positive disease. Collectively, these advances support a translational framework in which antibody therapy serves as a central component of treatment strategies, with targeted partners selected according to tumor biology and prior therapy. This review summarizes the biological basis, clinical evidence, and future perspectives of antibody-driven maintenance in HER2-positive metastatic breast cancer.

## 1. Introduction

Over the past two decades, the treatment landscape for human epidermal growth factor receptor 2–positive (HER2+) metastatic breast cancer (MBC) has changed markedly. Early therapeutic approaches relied on continuous chemotherapy combined with trastuzumab, reflecting the historical paradigm that cytotoxic therapy was essential for sustained disease control [1,2]. However, the CLEOPATRA trial established a new standard: a relatively short induction phase of dual HER2 blockade with trastuzumab and pertuzumab combined with a taxane (THP), followed by a maintenance phase with antibody therapy alone (HP) [3]. This trial demonstrated that sustained disease control can be achieved without the need for permanent chemotherapy, with antibody-based HER2-targeted therapy alone. In this study, patients received trastuzumab and pertuzumab until unacceptable toxicity or progression, combined with at least 6 three-weekly docetaxel cycles (median number of chemotherapy cycles was 8). The THP combination resulted in significant improvements in progression-free survival (PFS) and overall survival (OS) compared with trastuzumab plus docetaxel [3,4]. An exploratory analysis revealed that the optimal number of docetaxel cycles was 6, with more cycles having no additional impact on patients’ outcomes [5]. This paradigm shift reflects broader changes in systemic cancer therapy, driven by an improved understanding of tumor biology and resistance mechanisms.

Systemic therapy for MBC has evolved from predominantly cytotoxic approaches toward biologically driven strategies that aim to achieve durable disease control while minimizing cumulative toxicity. This shift reflects an improved understanding of tumor evolution, clonal heterogeneity, and adaptive resistance mechanisms, which challenge the long-term efficacy of continuous cytotoxic therapy. These advances have led to the development of maintenance strategies, in which biologically active agents are continued after induction chemotherapy, with the aim of long-term disease control and minimized toxicity. In contemporary practice, maintenance therapy may also involve the introduction of additional targeted agents aimed at reinforcing pathway suppression and prolonging disease control [6].

Building on this paradigm, CLEOPATRA now serves as the biological and clinical anchor for contemporary maintenance strategies. These data established a current standard of a defined induction phase followed by antibody-based continuation, a practice further reinforced by the PERTAIN trial, which supported antibody-based maintenance combined with endocrine therapy in HR+/HER2+ MBC [7,8]. Importantly, in contemporary clinical practice, patients with HR+/HER2+ disease typically receive endocrine therapy in combination with antibody-based continuation, whereas antibody therapy alone is rarely used in this subgroup. However, most patients eventually develop resistance to HER2-targeted therapy, highlighting the need to optimize these strategies beyond antibody therapy alone.

A few studies aimed at decreasing the intensity of the induction phase by using either less toxic chemotherapy or metronomic chemotherapy. Intravenous vinorelbine has been evaluated in a VELVET study (median number of vinorelbine cycles—9). The study demonstrated that vinorelbine can be safely combined with dual HER2 blockade and provided a clear clinical benefit [9]. Also, metronomic chemotherapy has been combined with dual HER2 blockade. In a multicenter, randomized, phase II study, elderly or frail, metastatic HER2+ patients received metronomic, oral cyclophosphamide (50 mg daily) combined with trastuzumab and pertuzumab. Addition of continuous, metronomic cyclophosphamide was well tolerated and was associated with a numerically longer median PFS by 7 months, resulting in a non-significant reduction in the risk of progression or death (hazard ratio [HR] for PFS = 0.65, *p* = 0.12) compared with dual HER2 blockade alone.

The shift from chemotherapy-dependent to antibody-driven disease control reflects a broader understanding of HER2 biology and mechanisms of resistance. Within this framework, sustained antibody-mediated suppression of the HER2 pathway may contribute to a state consistent with tumor dormancy, while Fc-mediated immune mechanisms facilitate anti-tumor immune surveillance [9]. In this context, efforts have focused on enhancing antibody-based maintenance therapy without compromising tolerability. [10]. Cyclin-dependent kinase 4 and 6 (CDK4/6) inhibitors and HER2 tyrosine kinase inhibitors (TKIs) have emerged as rational candidates, targeting cell-cycle progression and intracellular HER2 signaling, respectively, supported by accumulating biological and clinical evidence across different disease settings [11,12].

This narrative review examines the biological rationale and clinical evidence supporting antibody-based treatment strategies after chemotherapy in HER2+ MBC. We focus on recent pivotal trials, including PATINA and HER2-CLIMB-05, which evaluated targeted partners added to antibody therapy after induction therapy, as well as chemo-free proof-of-concept approaches such as monarcHER [13,14,15]. We also discuss emerging evidence from DETECT V, biomarker-driven treatment selection, and future directions toward personalized, chemotherapy-sparing strategies [16]. The goal of this review is to provide a translational framework that illustrates how antibody therapy serves as a central component of these strategies in HER2+ MBC.

## 2. Biological Rationale for Antibody-Driven Maintenance

Following the establishment of the clinical paradigm of induction chemotherapy followed by antibody-based maintenance, there is growing interest in the biological mechanisms that enable sustained disease control in this setting.

### 2.1. HER2 Pathway Suppression and Tumor Dormancy

The HER2 membrane receptor tyrosine kinase impacts tumor biology through multiple downstream pathways, including the phosphatidylinositol 3-kinase (PI3K)/AKT/mammalian target of rapamycin (mTOR) and RAS/RAF/mitogen-activated protein kinase (MEK)/extracellular signal-regulated kinase (ERK) signaling cascades [17]. Persistent activation of the HER2 pathway drives tumor cell proliferation, promotes survival, and contributes to resistance to apoptosis, underscoring the clinical importance of sustained HER2 suppression for long-term disease control [18]. Trastuzumab and pertuzumab bind to different epitopes within the extracellular domain of HER2, thereby interfering with receptor dimerization and downstream signaling. Compared with single-agent trastuzumab, dual HER2 blockade provides more robust pathway inhibition and limits compensatory signaling through human epidermal growth factor receptor 3 and related escape mechanisms [19]. In hormone receptor-positive disease, bidirectional crosstalk between estrogen receptor (ER) and HER2 signaling pathways further contributes to adaptive resistance, providing a biological rationale for combined targeting strategies [20].

Antibody-mediated suppression of HER2 signaling may contribute to sustained disease control through multiple mechanisms, including direct anti-proliferative effects and induction of apoptosis, and in some cases may also promote a state consistent with tumor dormancy, in which residual cancer cells persist without active proliferation, although clinical responses may also include complete remission or deep, durable disease control [21]. This state of reduced tumor activity appears to be maintained by continuous antibody exposure that limits HER2 receptor activation and downstream signaling [22]. Consistent with this concept, preclinical models have shown that withdrawal of HER2-targeted therapy is followed by rapid tumor regrowth, reflecting ongoing treatment-dependent disease control rather than a stable dormant state [23].

The concept of tumor dormancy has important implications for maintenance strategies. Rather than requiring continuous cytotoxic therapy to suppress proliferation, antibody-driven HER2 blockade may contribute to sustained disease control through a combination of pathway inhibition, cytotoxic effects, and immune-mediated mechanisms, with tumor dormancy representing one potential contributing mechanism [24]. This strategy limits cumulative chemotherapy-related toxicity while maintaining quality of life, which is particularly important for patients requiring a chronic palliative systemic treatment [25].

### 2.2. Fc-Mediated Immune Effector Functions

Beyond direct pathway inhibition, trastuzumab and pertuzumab engage the immune system through Fc-mediated effector functions, including antibody-dependent cellular cytotoxicity (ADCC) and antibody-dependent cellular phagocytosis (ADCP) [26]. In addition to NK cell–mediated ADCC, macrophage-driven ADCP is increasingly recognized as a major mechanism contributing to the activity of HER2-targeted antibodies. The Fc region of these antibodies binds to Fcγ receptors on natural killer cells, macrophages, and other immune effector cells, leading to tumor cell lysis and phagocytosis [27]. Genetic polymorphisms in Fcγ receptor genes, particularly *FCGR2A* and *FCGR3A*, affect ADCC and have been linked to variability in clinical outcomes among patients treated with trastuzumab [28].

Immune-mediated mechanisms appear to contribute to the durability of antibody-based maintenance strategies. Preclinical studies indicate that trastuzumab-mediated ADCC is enhanced by the addition of pertuzumab, consistent with synergistic immune activation achieved through dual HER2 blockade [29]. Importantly, the efficacy of antibody-mediated immune mechanisms may be influenced by the preceding induction phase, as chemotherapy-induced tumor cell death can promote antigen release, enhance antigen presentation, and stimulate immune activation, thereby creating a more favorable microenvironment for subsequent antibody-dependent effector functions [30]. Clinical observations across multiple settings indicate that patients with favorable Fcγ receptor genotypes may experience enhanced outcomes with trastuzumab-based therapy, supporting a role for immune effector functions in antibody-driven disease control [31]. The role of immune mechanisms in the efficacy of HER2-targeting antibodies has been demonstrated in the phase III SOPHIA trial, which evaluated margetuximab, a trastuzumab-based antibody containing a modified Fc region to increase affinity for Fcγ receptors. In this study, margetuximab plus chemotherapy compared to trastuzumab plus chemotherapy significantly improved PFS in pretreated HER2-positive advanced breast cancer patients [32].

The importance of Fc-mediated immunity extends to continuation strategies that combine antibodies with targeted partners. CDK4/6 inhibitors and HER2 TKIs are not known to interfere with antibody Fc function, allowing immune effector mechanisms to remain active during combination therapy [33,34]. This preservation of immune engagement distinguishes antibody-based maintenance from standard, intravenous chemotherapy-based approaches, which may suppress immune function through myelosuppression, leucopenia, and lymphopenia [35]. Together, the preservation of immune effector function provides an additional biological rationale for antibody-based therapy strategies following chemotherapy. These mechanisms align with broader efforts to leverage immune-mediated effects in cancer therapy. Importantly, increasing understanding of immune signaling pathways and immune-target discovery provides a mechanistic framework for integrating antibody-mediated immunity with emerging immunotherapeutic strategies.

## 3. Antibody-Based Maintenance After Chemotherapy

### 3.1. Rationale for Optimizing Antibody Maintenance After Induction Chemotherapy

While continuation of antibody-based therapy after induction chemotherapy is an established strategy in HER2+ MBC, most patients eventually experience disease progression on antibody maintenance alone, highlighting the limitations of sustained HER2 blockade as a single modality [36]. This pattern of progression suggests that residual proliferative signaling and adaptive resistance mechanisms facilitate tumor escape despite ongoing HER2 suppression. Consequently, clinical efforts have focused on optimizing antibody therapy by adding targeted, non-cytotoxic partners that enhance disease control without the need to continue prolonged intravenous chemotherapy.

The biological mechanisms described above provide a direct rationale for the design of contemporary maintenance strategies evaluated in clinical trials. In particular, combining extracellular HER2 blockade with targeted agents acting on complementary pathways allows for more comprehensive suppression of tumor cell survival and proliferation, thereby supporting durable disease control. CDK4/6 inhibitors reinforce control of cell-cycle progression, whereas HER2 tyrosine kinase inhibitors provide intracellular inhibition of HER2 signaling, complementing antibody-mediated blockade at the cell surface and enabling multi-level suppression of oncogenic signaling. Importantly, this approach differs from traditional chemotherapy-based combinations, as it is based on targeted, non-cytotoxic mechanisms. For example, agents such as CDK4/6 inhibitors are associated with a high incidence of neutropenia, but this reflects reversible cell-cycle arrest rather than cytotoxic damage to bone marrow precursors, distinguishing it mechanistically from chemotherapy-induced myelosuppression and resulting in a lower risk of clinically significant complications such as febrile neutropenia [37].

### 3.2. PATINA: CDK4/6 Inhibition Plus Dual HER2 Blockade

The PATINA trial (AFT-38) represents the first randomized phase III evaluation of CDK4/6 inhibitor-based maintenance therapy in hormone receptor-positive (HR+)/HER2+ MBC. This open-label study included 518 patients who had completed 6–8 cycles of first-line induction therapy with trastuzumab, with or without pertuzumab, in combination with a taxane or vinorelbine, and had no evidence of disease progression. Patients were randomized 1:1 to receive palbociclib (125 mg once daily, days 1–21 of a 28-day cycle) in combination with trastuzumab ± pertuzumab and endocrine therapy, or antibody plus endocrine therapy alone [15].

In the primary analysis, PFS was significantly longer with the addition of palbociclib to antibody-based therapy. Median PFS was 44.3 months in the palbociclib arm compared with 29.1 months in the control arm, representing a substantial absolute difference, with a hazard ratio of 0.74 (95% confidence interval [CI] 0.58–0.94), indicating a moderate relative reduction in the risk of progression or death [15]. This magnitude of benefit appears numerically greater than that reported for some prior maintenance strategies in HER2+ MBC, and supports the role of CDK4/6 inhibition in reinforcing antibody-driven disease control, without the need for continuous chemotherapy.

The PATINA design aligns with the CLEOPATRA paradigm of short-term chemotherapy induction followed by antibody continuation, extending this framework by integrating targeted cell-cycle inhibition. Subgroup analyses showed broadly similar effects across predefined patient subgroups, including those defined by prior anti-HER2 therapy and induction treatment response [15]. Although some confidence intervals crossed unity, the consistency of effect supports a generally consistent benefit across subgroups rather than an effect limited to selected populations.

The safety profile of PATINA was consistent with the known toxicities of palbociclib and HER2-directed therapy. Grade 4 adverse events occurred in 10.0% of patients receiving palbociclib compared with 3.6% in the control arm. Neutropenia was the most frequent adverse event (AE) associated with palbociclib, occurring in 77.8% of patients (grade 3 (G3): 60.5%; grade 4: 4.6%), compared with 7.7% in the control arm. Other common G ≥ 2 AEs included diarrhea (37.2%), fatigue (27.2%), and stomatitis (9.9%). Treatment discontinuation due to adverse events occurred in 18.0% of palbociclib-treated patients, representing a clinically relevant proportion, with no treatment-related deaths reported [15].

Patient-reported outcome analyses indicated that the addition of palbociclib was not associated with a deterioration in health-related quality of life, despite higher rates of hematologic toxicity and treatment discontinuation. Time to worsening of patient-reported symptoms was comparable across treatment groups, and global quality-of-life scores remained stable over extended follow-up [38]. These findings highlight the importance of balancing efficacy with treatment tolerability in the maintenance setting. In addition to the observed PFS benefit, these data support a favorable benefit-risk profile for palbociclib plus dual HER2 blockade and endocrine therapy as a continuation strategy following induction chemotherapy in HR+/HER2+ MBC.

### 3.3. HER2 Tyrosine Kinase Inhibitors as Maintenance Partners: HER2-CLIMB-05

The exploration of HER2 TKIs as partners for antibody-based therapy began in later-line settings, where lapatinib and tucatinib have been combined with trastuzumab, with or without chemotherapy. In a phase III study, the combination of lapatinib and trastuzumab in HER2+ MBC patients pretreated with various trastuzumab-based therapies (median 3 lines), significantly improved PFS (HR = 0.73; 95% CI, 0.57 to 0.93) and showed a numerical improvement in OS (HR = 0.75; 95% CI, 0.53 to 1.07) compared to lapatinib alone [39]. In the HER2-CLIMB study, a combination of tucatinib with trastuzumab and capecitabine significantly improved PFS (HR = 0.54; 95%CI 0.42 to 0.71) and OS (HR = 0.73; 95%CI 0.59–0.90) compared with trastuzumab plus capecitabine [40,41]. Building on this conceptual framework, the HER2-CLIMB-05 study was designed to evaluate whether selective intracellular HER2 inhibition could reinforce antibody-based therapy after induction chemotherapy in the first-line setting.

The HER2-CLIMB-05 trial was a randomized, double-blind, placebo-controlled phase III study in which tucatinib or placebo was added to trastuzumab and pertuzumab as first-line maintenance therapy in 654 patients with HER2+ MBC. Eligible patients had centrally confirmed HER2+, metastatic or unresectable, locally advanced disease and had completed 4–8 cycles of induction therapy with trastuzumab, pertuzumab, and a taxane without evidence of disease progression. Patients with asymptomatic brain metastases were allowed to enroll.

Primary results presented recently demonstrated a statistically significant improvement in PFS with tucatinib-based maintenance. Median PFS was 24.9 months in the tucatinib arm compared with 16.3 months in the placebo arm, corresponding to an absolute improvement of 8.6 months and an HR for PFS of 0.641 (95% CI 0.514–0.799). Differences in patient populations between HER2-CLIMB-05 and trials such as PATINA, including the inclusion of patients with HR-negative disease and brain metastases in HER2-CLIMB-05, may partially explain the shorter PFS observed in the control arm. Consistent PFS benefit was observed across prespecified subgroups, including patients with and without brain metastases and irrespective of HR status. Overall survival data were immature at the time of the primary analysis [13].

HER2-CLIMB-05 applies a multi-level HER2 targeting strategy by combining dual extracellular blockade with trastuzumab and pertuzumab with selective intracellular inhibition of HER2 kinase activity by tucatinib. This approach is designed to address resistance mechanisms that may arise during antibody-based continuation, including incomplete receptor blockade, *HER2*-activating mutations, downstream pathway reactivation, and compensatory signaling through other receptor tyrosine kinases [42].

Tucatinib is a highly selective HER2 TKI with minimal off-target inhibition of the epidermal growth factor receptor, distinguishing it from earlier HER2-directed TKIs and contributing to a favorable tolerability profile [43]. By inhibiting intracellular HER2 signaling, tucatinib complements the extracellular inhibition achieved by trastuzumab and pertuzumab, providing a coherent biological rationale for sustained HER2 pathway suppression during maintenance therapy [44]. Given the central role of Fc-mediated immune mechanisms in antibody-based strategies, intracellular HER2 inhibition may also influence receptor availability at the cell surface; however, the clinical efficacy observed in HER2-CLIMB-05 suggests that these interactions do not compromise the overall therapeutic activity.

The safety profile observed in HER2-CLIMB-05 was consistent with the known toxicities of tucatinib when used in combination with dual HER2-directed antibody therapy. Grade ≥ 3 adverse events were more frequent in the tucatinib than in the placebo arm (42.3% vs. 24.4%). Diarrhea was the most commonly reported AE, occurring in 72.7% of patients receiving tucatinib (G ≥ 3: 6.1%) compared with 51.2% in the control arm (G ≥ 3: 4.0%). Elevations in liver transaminases were more frequent with tucatinib, including G ≥ 3 alanine aminotransferase increases in 13.5% and aspartate aminotransferase increases in 7.1% of patients. Treatment discontinuation due to AEs occurred in 13.8% of patients receiving tucatinib and in 4.6% of patients in the placebo arm. No new safety signals were identified [13]. While tucatinib-based strategies avoid the use of conventional cytotoxic chemotherapy, they are associated with a distinct toxicity profile, and the overall treatment burden should be considered in the context of maintenance therapy.

Collectively, HER2-CLIMB-05 supports tucatinib plus trastuzumab and pertuzumab as an effective maintenance strategy following induction chemotherapy in HER2+ MBC. In contrast to continuation strategies based on CDK4/6 inhibition, which are biologically most relevant in HR+ disease, tucatinib-based maintenance showed activity regardless of HR status. Together with CDK4/6 inhibitor-based strategies, as shown by the PATINA trial, HER2-CLIMB-05 illustrates complementary approaches to extending antibody-driven disease control by incorporating targeted, non-chemotherapy partners after initial induction therapy.

The key clinical and translational studies underpinning antibody-based maintenance strategies in HER2+ MBC are summarized in Table 1.

## 4. Chemotherapy-Free Antibody-Based Strategies: Biological Proof-of-Concept

Antibody-based treatment strategies in HER2+ breast cancer can be divided into two complementary approaches: escalation strategies, as exemplified by trials such as PATINA, which reinforce antibody continuation with targeted agents, and de-escalation strategies, which aim to replace chemotherapy with biologically driven combinations.

### 4.1. CDK4/6 Inhibition Without Chemotherapy: monarcHER

In this context, chemotherapy-free strategies represent a de-escalation approach aimed at replacing cytotoxic therapy with targeted combinations. The monarcHER trial provided a translational proof-of-concept supporting combined HER2 and CDK4/6 inhibition in HR+/HER2+ MBC in a treatment-refractory setting rather than in a maintenance context. In this phase II study, 237 patients were randomized to one of three treatment arms: abemaciclib plus trastuzumab plus fulvestrant, abemaciclib plus trastuzumab, or chemotherapy plus trastuzumab. The study population was heavily pretreated, with obligatory prior exposure to trastuzumab and pertuzumab, and up to 2 prior lines of HER2-targeted therapies in the metastatic setting [14].

The primary endpoint, investigator-assessed PFS, favored the combination of abemaciclib, trastuzumab, and fulvestrant over chemotherapy plus trastuzumab, with a median PFS of 8.3 vs. 5.7 months (HR 0.67; *p* = 0.051, not reaching conventional statistical significance but meeting the prespecified endpoint within the trial design). The abemaciclib plus trastuzumab arm without endocrine therapy achieved a median PFS of 5.7 months, which did not demonstrate superiority over the chemotherapy-containing arm, highlighting the limited efficacy of the abemaciclib–trastuzumab doublet in the absence of endocrine therapy [14]. In contrast, the improved outcomes observed with the triplet combination underscore the importance of concurrent endocrine therapy in this setting. With longer follow-up, overall survival numerically favored abemaciclib-based combinations compared with chemotherapy plus trastuzumab [47].

Importantly, monarcHER was not designed to redefine clinical practice but to explore the biological feasibility of combining HER2-directed antibodies with CDK4/6 inhibition in a challenging, treatment-refractory population. In this context, the observed PFS signal, although modest, primarily driven by the triplet combination demonstrated that cell-cycle inhibition could reinforce antibody-driven disease control in HR+/HER2+ tumors, where bidirectional crosstalk between ER and HER2 signaling pathways is a recognized mechanism of therapeutic resistance [20], as discussed in the biological rationale section.

Exploratory biomarker analyses further supported this biological interpretation. Patients with luminal intrinsic subtypes, as defined by PAM50, derived greater benefit from abemaciclib-based regimens than those with HER2-enriched tumors [47]. This finding is consistent with the partial dependence of HR+/HER2+ luminal tumors on ER-driven cell-cycle progression, contributing to sensitivity to CDK4/6 inhibition [48,49]. These observations provided a strong rationale for subsequent trials evaluating CDK4/6 inhibitors as partners for antibody-based strategies in earlier disease settings.

The translational significance of monarcHER thus extends beyond its immediate clinical outcomes. The confirmation that disease control can be achieved with combined antibody and CDK4/6 inhibitor therapy in the absence of chemotherapy established a framework for antibody-based strategies centered on targeted pathway inhibition rather than cytotoxic therapy. This proof-of-concept supports the development of chemotherapy-free maintenance strategies combining CDK4/6 inhibitors with dual HER2 blockade and endocrine therapy in HR+/HER2+ MBC [14,15,40].

### 4.2. Biomarker-Driven Sensitivity: The PATRICIA Trial

The SOLTI-1303-PATRICIA trial further refined the biological rationale for chemotherapy-free strategies by demonstrating that sensitivity to combined HER2 and CDK4/6 inhibition is strongly influenced by intrinsic tumor biology. This investigator-initiated, multicenter, phase II study evaluated palbociclib in combination with trastuzumab, with or without endocrine therapy, in 71 patients with heavily pretreated HR+/HER2+ MBC [45].

Patients were required to have received trastuzumab-based therapy and have undergone 2 to 4 prior lines of systemic treatment in the metastatic setting. Patients were analyzed in three cohorts: A (ER-negative treated with trastuzumab plus palbociclib), B1 (ER-positive treated with trastuzumab plus palbociclib without endocrine therapy), and B2 (ER-positive treated with trastuzumab plus palbociclib and letrozole). At a median follow-up of 60.5 months, the median OS for the entire population was 29.8 months. Four-year OS rates differed substantially across cohorts, reaching 35.7% in cohort B1 and 32.3% in cohort B2, compared with 13.3% in cohort A [45].

Exploratory molecular analyses identified the PAM50 intrinsic subtype as a key predictor of long-term outcomes. Patients with luminal subtypes experienced significantly longer PFS compared with non-luminal tumors (hazard ratio 0.48, 95% CI 0.24–0.96), with a numerically longer median OS (38.0 vs. 26.8 months). Multivariable analyses confirmed PAM50 luminal status as the only factor associated with improved PFS after adjustment for clinical variables [45].

Gene expression profiling supported this interpretation. Higher expression of luminal-associated genes and chemoendocrine signatures was associated with more favorable long-term outcomes, whereas basal-like and proliferation-related gene signatures were associated with treatment resistance [45]. These observations highlight the role of ER-driven signaling dependency and cell-cycle dependence in shaping responses to CDK4/6-based combinations, as discussed previously.

Additional support for biomarker-guided strategies came from PATRICIA cohort C, in which palbociclib combined with trastuzumab and endocrine therapy demonstrated superior PFS compared with the physician’s treatment of choice in patients with ER+ PAM50 luminal tumors [45]. Together, these findings suggest that intrinsic molecular subtype provides important biological context beyond hormone receptor status alone; however, in routine clinical practice, treatment decisions remain primarily guided by hormone receptor status assessed by immunohistochemistry.

Collectively, the PATRICIA trial provides translational evidence that chemotherapy-free antibody-based strategies may be effective in biologically defined subsets of HR+/HER2+ disease. These findings support the potential role of biomarker-driven approaches for selecting targeted partners within antibody-centered treatment paradigms.

### 4.3. Chemotherapy De-Escalation and CDK4/6 Integration: The DETECT V Trial

The phase III DETECT V trial suggests that chemotherapy may be omitted in selected patients with HR+/HER2+ MBC when dual HER2 blockade is combined with endocrine therapy. In this multicenter study, 262 HR+/HER2+ MBC patients were randomized to first-line treatment based either on a chemotherapy-free regimen (trastuzumab, pertuzumab, endocrine therapy, with ribociclib introduced as a protocol amendment and therefore not part of the original randomized design) or chemotherapy-containing regimen (trastuzumab, pertuzumab, chemotherapy followed by maintenance with dual anti-HER2 blockade plus ET [±ribociclib according to the amended protocol]) [16].

The trial met its primary endpoint of improved treatment tolerance. The chemotherapy-free arm was associated with lower overall toxicity than the chemotherapy-containing arm (modified AE score: 52.3% vs. 76.9%, *p* < 0.001), without a statistically significant difference in efficacy. Median PFS was 19.5 months in the chemotherapy-free arm and 23.0 months in the chemotherapy-containing arm (HR 1.15, 95% CI 0.82–1.62), with no statistically significant difference in OS (median not reached vs. 46.1 months; HR 0.98, *p* = 0.928) [16]. These findings indicate that effective dual HER2 blockade combined with endocrine therapy can provide comparable disease control without a statistically significant difference compared with chemotherapy-based regimens, while markedly reducing treatment-related toxicity.

It is noteworthy that the inclusion of ribociclib in the DETECT V protocol was amended after the initial enrolment of 124 patients. Importantly, ribociclib was not incorporated as part of the original randomized comparison but was introduced in subsequent cohorts, primarily within the chemotherapy-free arm. In a non-randomized comparison of subsequent cohorts, ribociclib addition was associated with a substantial improvement in clinical outcomes. Median PFS was 29.7 months in patients receiving ribociclib compared with 15.6 months in those without ribociclib (HR 0.48, 95% CI 0.34–0.67). Median OS was not reached in the ribociclib cohort compared to 46.1 months in patients treated without ribociclib (HR 0.43, 95% CI 0.26–0.72). Multivariable analyses confirmed ribociclib treatment as an independent predictor of improved PFS (HR 0.49, *p* < 0.001) and OS (HR 0.44, *p* = 0.002) [16].

From a translational perspective, DETECT V extends the biological framework established by monarcHER and PATRICIA into the first-line setting. The randomized component confirms that sustained HER2 pathway suppression combined with endocrine therapy can substitute for classic cytotoxic chemotherapy in HR+/HER2+ disease. At the same time, the ribociclib amendment suggests a potential additional benefit in antibody-based disease control. The contribution of ribociclib should, however, be interpreted with caution, as its use was introduced as a protocol amendment and not evaluated within the original randomized design. These findings support the biological rationale for integrating CDK4/6 inhibitors into chemotherapy-free, antibody-centered strategies, while prospective validation remains warranted. Notably, this approach differs conceptually from maintenance strategies such as PATINA, as it represents a chemotherapy-free model without a distinct induction phase, in which patients receive from the outset a combination of anti-HER2 therapy and endocrine therapy, with optional CDK4/6 inhibition, mirroring the antibody-based treatment approach typically used after induction in conventional paradigms.

### 4.4. TOUCH: Neoadjuvant Translational Evidence Supporting Chemotherapy-Free HER2/CDK4/6 Strategies

The TOUCH trial (IBCSG 55-17) provides additional translational evidence supporting the replacement of chemotherapy with CDK4/6 inhibitors when combined with dual HER2 blockade in HR+/HER2+ breast cancer. This randomized phase II neoadjuvant study enrolled 147 postmenopausal patients with HR+/HER2+ early breast cancer and compared a taxane-based regimen (weekly paclitaxel plus trastuzumab and pertuzumab) with a chemotherapy-free regimen consisting of palbociclib, letrozole, trastuzumab, and pertuzumab administered for 16 weeks [46].

Pathological complete response (pCR) rates were comparable between the two treatment arms, reaching 32.9% (95% CI 22.3–44.9) with paclitaxel plus dual HER2 blockade and 33.3% (95% CI 22.7–45.4) with the palbociclib-based chemotherapy-free regimen. Importantly, treatment completion was higher in the palbociclib arm (94.4% vs. 79.5%), highlighting the feasibility and safety of neoadjuvant dual HER2-blockade combined with CDK4/6i [45].

Exploratory molecular analyses further strengthened the biological rationale for this approach. PAM50 intrinsic subtyping showed that non-luminal tumors achieved the highest pCR rates (45.5%) regardless of treatment backbone, consistent with the known sensitivity of this subtype to HER2 pathway inhibition. In contrast, luminal tumors exhibited lower pCR rates overall; however, the 18.4% pCR observed in luminal cancers treated with palbociclib, endocrine therapy, and dual HER2 blockade appeared higher than previously reported results from neoadjuvant studies using endocrine therapy and anti-HER2 agents without CDK4/6 inhibition, although such comparisons should be interpreted with caution given differences in study design and patient populations [45]. Importantly, these pCR rates remain lower than those achieved with standard chemotherapy-containing regimens [50,51]. This finding suggests that CDK4/6 blockade may contribute to overcoming endocrine resistance in luminal HER2+ disease by disrupting ER–HER2 crosstalk and cell-cycle dependency.

Although TOUCH was conducted in the neoadjuvant setting and was not designed to inform treatment sequencing in metastatic disease, its results provide important translational validation that tumor responses can be achieved without cytotoxic chemotherapy through a combination of HER2 pathway suppression and cell-cycle inhibition. Together with metastatic trials such as monarcHER and PATRICIA, the TOUCH study supports a broader biological concept that chemotherapy-free antibody-based strategies are mechanistically sound and clinically feasible, forming a key component of evolving targeted and de-escalation strategies in HR+/HER2+ breast cancer. These findings also highlight the need for refined patient selection and prospective validation across different disease settings.

## 5. Future Perspectives and Unanswered Questions

### 5.1. Biomarker-Driven Treatment Selection

The biological heterogeneity of HR+/HER2+ breast cancer underscores the need for biomarker-driven approaches to optimize antibody-based treatment strategies [48,49]. Growing evidence indicates that treatment benefit in this subtype is influenced not only by HR expression, but also by intrinsic tumor characteristics and signaling dependencies [52]. Importantly, the sensitivity of luminal HR+/HER2+ tumors to chemotherapy-free strategies is likely dependent on a functional estrogen receptor axis, which can be effectively targeted by endocrine therapy, whereas endocrine resistance mechanisms, including ESR1 mutations, may limit the efficacy of such approaches [53]. Advances in integrative molecular profiling have further highlighted the complexity of oncogenic signaling networks, emphasizing that HER2 represents only one dimension of tumor biology. Multi-omic approaches integrating genomic, transcriptomic, and proteomic data may provide a more comprehensive understanding of pathway dependencies and support the rational development of combination strategies targeting multiple oncogenic drivers [54,55].

PAM50 intrinsic subtyping is one of the most informative biomarkers for stratifying HR+/HER2+ disease in translational research settings [56]. Tumors with HER2-enriched biology are characterized by dominant HER2 pathway activation and relative independence from ER signaling, which renders them highly sensitive to HER2-targeted therapy alone [57]. In contrast, luminal HER2+ tumors retain functional ER signaling and exhibit pronounced ER-HER2 crosstalk, contributing to adaptive resistance to HER2 blockade [20]. These features provide a strong rationale for incorporating CDK4/6 inhibitors and endocrine treatment into antibody-based strategies in luminal disease. However, in routine clinical practice, treatment decisions are primarily guided by hormone receptor status assessed by immunohistochemistry. Additionally, PAM50 subtyping is typically performed on primary tumors, and subtype conversion during disease progression may require reassessment of metastatic tissue.

Translational data from multiple clinical settings suggest that CDK4/6 inhibitor-based combinations may be particularly effective in tumors with luminal intrinsic biology. HER2-enriched tumors may also derive benefit from intensified HER2 pathway suppression; however, the optimal treatment approach in this subgroup remains to be defined [46]. Importantly, clinical trial data do not currently support restricting the benefit of CDK4/6 inhibition to specific intrinsic subtypes. Collectively, these observations suggest that PAM50 subtyping provides biological context that may inform, but does not currently determine, the selection of targeted partners for antibody-based strategies [40,45,47,58]. Similarly, although HER2 TKIs represent a biologically plausible strategy for tumors with HER2-enriched features, clinical trial data to date have not demonstrated a subtype-specific benefit, and their optimal positioning remains to be defined.

Beyond intrinsic subtype, additional biomarkers may further refine treatment selection. Alterations in the PI3K/AKT pathway, including PIK3CA mutations and PTEN loss, have been associated with resistance to HER2-directed antibodies and may identify tumors requiring more comprehensive HER2 pathway suppression [59,60]. In particular, PIK3CA mutations may enable downstream signaling independent of receptor-level inhibition, providing a rationale for selecting intracellular inhibitors, such as HER2 TKIs or PI3K inhibitors, in selected contexts over antibody-based strategies alone. Although therapeutic targeting of this axis has been limited by toxicity, these biomarkers remain of interest as predictors of differential sensitivity to antibody-based strategies augmented by intracellular pathway inhibitors [61,62].

Host-related factors may also influence the efficacy of antibody-based strategies. In particular, polymorphisms in Fcγ receptors can influence ADCC and phagocytosis and have been associated with variability in clinical outcomes among patients receiving trastuzumab-based regimens [28,31]. While not yet validated for clinical decision-making, Fcγ receptor genotypes may represent a potential biomarker of response; however, these biomarkers are not currently standardized, clinically available, or actionable for treatment selection.

Taken together, these considerations highlight that future maintenance and chemotherapy-free strategies in HR+/HER2+ breast cancer will likely require integrated biomarker assessment that combines intrinsic tumor subtype, pathway activation profiles, and host immune factors. Prospective validation will be required to support the use of biomarker-driven strategies in personalized antibody-centered treatment.

### 5.2. De-Escalation Strategies and Chemotherapy-Free Approaches

The success of antibody-based maintenance strategies raises the question of whether chemotherapy can be omitted entirely in selected patients. Translational and early clinical data suggest that deep tumor responses may be achievable through combined HER2 pathway suppression and cell-cycle inhibition without cytotoxic therapy, supporting the biological feasibility of chemotherapy-free approaches in HR+/HER2+ disease, particularly for patients in whom chemotherapy toxicity is a major concern [63].

Several important questions remain unanswered. First, whether chemotherapy can be safely omitted in broader patient populations remains uncertain, although studies such as DETECT V suggest that chemotherapy-free approaches may be feasible in carefully selected populations. The CLEOPATRA paradigm established that a short chemotherapy induction phase followed by antibody therapy is highly effective, but whether antibody-based combinations with targeted partners can achieve comparable long-term outcomes without chemotherapy remains to be determined [36].

Second, optimal treatment sequencing after progression on antibody-based maintenance has yet to be defined. Highly active antibody–drug conjugates (ADCs), including trastuzumab deruxtecan, have reshaped later-line treatment of HER2+ MBC [64]. Whether prior exposure to CDK4/6 inhibitors or HER2 TKIs during treatment influences subsequent sensitivity to ADCs represents an important unanswered clinical question that warrants prospective evaluation. In particular, prolonged HER2 pathway suppression, especially with HER2 TKIs, may modulate HER2 receptor expression and cellular localization, potentially impacting the efficacy of subsequent HER2-targeted ADCs [65].

Third, the possibility of treatment discontinuation in patients achieving prolonged complete responses on antibody-based therapy remains unexplored. Although deep tumor responses may be achieved, these do not necessarily translate into durable disease control, which is more appropriately reflected by progression-free and overall survival outcomes. In other malignancies, including chronic myeloid leukemia, treatment-free intervals have been successfully introduced in patients who achieve complete molecular responses; however, this comparison is conceptual, as fundamental biological differences limit direct clinical extrapolation [66]. Whether similar strategies can be applied in HER2+ MBC, and which biomarkers might predict durable disease control after treatment interruption, remain areas of active investigation [67].

### 5.3. Integration with Novel HER2-Targeted Agents

The ongoing development of novel HER2-targeted agents is expected to reshape future induction and maintenance strategies in HER2+ MBC. Bispecific antibodies, ADCs, and next-generation HER2 TKIs introduce new mechanisms of HER2 pathway suppression that may ultimately influence how antibody-based approaches are conceptualized and applied [68].

Bispecific antibodies, such as zanidatamab, which simultaneously bind two distinct HER2 epitopes, have demonstrated promising antitumor activity and greater receptor engagement than single-epitope antibodies [69]. This biparatopic binding (to distinct extracellular domains of HER2) promotes receptor clustering, internalization, and enhanced HER2 downregulation compared with conventional antibody approaches. From a translational perspective, these agents may offer enhanced and sustained HER2 pathway inhibition, raising the possibility that bispecific antibodies could serve as future key components of antibody-based strategies, either alone or in combination with CDK4/6 inhibitors, HER2 TKIs, or downstream pathway inhibitors. However, their role in long-term disease control and maintenance settings remains to be defined [70].

ADCs represent a distinct therapeutic class by coupling HER2 targeting with intracellular delivery of cytotoxic payloads. Trastuzumab deruxtecan has demonstrated marked activity in later-line HER2+ MBC treatment, leading to its evaluation in earlier lines [64]. The phase III DESTINY-Breast09 trial directly addresses whether an ADC-based regimen can replace trastuzumab, pertuzumab, and chemotherapy as first-line induction therapy [71]. Emerging data suggest that these approaches represent a separate therapeutic paradigm, with continuous treatment until progression or unacceptable toxicity, without a defined induction or distinct treatment phases. As the cytotoxic payload is intrinsically linked to the antibody, these regimens do not allow for de-escalation to antibody-only therapy, in contrast to the CLEOPATRA model.

Next-generation HER2 TKIs with improved selectivity and pharmacologic properties are also being studied [72]. While agents such as neratinib have demonstrated activity in combination with chemotherapy in MBC and in extended adjuvant settings, their optimal role in antibody-based maintenance strategies in palliative treatment remains uncertain. This is particularly relevant given their gastrointestinal toxicity profile, including a high incidence of grade ≥3 diarrhea requiring proactive management, which may limit their suitability in a maintenance phase focused on minimizing cumulative toxicity and preserving quality of life [73,74]. Future studies should determine whether TKIs are best used as partners in antibody-based strategies, as part of combination induction regimens, or in later treatment lines after exposure to antibodies or ADCs.

Overall, incorporating emerging HER2-targeted agents into treatment strategies will require careful attention to treatment sequencing, biological complementarity, and biomarker-guided patient selection. As induction paradigms continue to evolve, a central challenge will be to preserve the core principle established by the CLEOPATRA model—durable disease control through sustained HER2 suppression—while minimizing cumulative toxicity and maintaining quality of life, as reflected in maintenance-focused trials such as PATINA and HER2-CLIMB-05.

Interpretation of the available data is limited by heterogeneity across studies, the exploratory nature of biomarker analyses, and immature overall survival results, including in key trials such as PATINA and HER2-CLIMB-05.

## 6. Conclusions

The management of HER2+ MBC has shifted from continuous chemotherapy-based regimens toward a model of a short chemotherapy induction phase followed by sustained antibody-driven disease control. The CLEOPATRA paradigm established the dual HER2 blockade as the biological and clinical foundation of this maintenance strategy, demonstrating that ongoing disease suppression can be preserved through long-term antibody administration rather than continuous cytotoxic therapy. Building on this framework, recent trials have validated the concept that antibody-based approaches can be effectively enhanced by combining them with targeted partners, such as CDK4/6 inhibitors or HER2 TKIs, to maintain disease control with minimal impact on quality of life. At the same time, emerging evidence from chemotherapy-free approaches suggests that, in selected patients, effective disease control may be achieved without a distinct induction phase, supporting alternative treatment models that may not be applicable across all clinical settings.

Collectively, evidence from PATINA, HER2-CLIMB-05, and many biologically informative studies supports a translational model in which antibody continuation serves as the backbone of maintenance strategies, with partner selection guided by tumor biology. However, biomarkers such as intrinsic subtyping should currently be regarded as research tools rather than determinants of treatment selection, and their clinical utility requires prospective validation. As new HER2-targeted agents continue to influence initial therapeutic strategies, including ADC-based approaches that may challenge the traditional induction–continuation paradigm, future research will need to address optimal treatment sequencing, the feasibility of de-escalation or treatment interruption, and the systematic incorporation of patient-reported outcomes. Taken together, these developments point toward treatment strategies that are durable, biologically grounded, and aligned with patient-centered care in HER2+ MBC.

## Figures and Tables

**Table 1:** Antibody-based and targeted strategies in HER2-positive breast cancer: key clinical and translational studies.

Trial	Phase	Clinical Setting	Population	Induction/Backbone	Maintenance/Combination Strategy	Therapeutic Concept	Key Translational Message
CLEOPATRA [4]	III	MBC (1L)	HER2+ (all HR)	Docetaxel + trastuzumab + pertuzumab	Trastuzumab + pertuzumab	Dual HER2 blockade	Established finite chemotherapy induction followed by antibody continuation as standard paradigm
PERTAIN [7]	II	MBC (1L)	HR+/HER2+	Endocrine therapy + trastuzumab ± pertuzumab (± taxane at investigator discretion)	ET + anti-HER2 antibodies	Endocrine–HER2 platform	Defined endocrine therapy plus dual HER2 blockade as a viable antibody-based backbone in HR+ disease
PATINA (AFT-38) [15]	III	MBC (1L, post-induction maintenance)	HR+/HER2+	Taxane or vinorelbine + trastuzumab ± pertuzumab	Palbociclib + ET + trastuzumab ± pertuzumab	Maintenance escalation	Validated CDK4/6 inhibition as a maintenance partner on an antibody backbone
HER2-CLIMB-05 [13]	III	MBC (1L, post-induction maintenance)	HER2+ (all HR)	Taxane + trastuzumab + pertuzumab	Tucatinib + trastuzumab + pertuzumab	HER2 pathway intensification	Demonstrated sustained multi-agent HER2 suppression as maintenance strategy
monarcHER [14]	II	MBC (later-line, treatment-refractory)	HR+/HER2+ (pretreated)	Trastuzumab-based	Abemaciclib + trastuzumab ± fulvestrant	Chemo-free proof-of-concept	Showed antibody + ET + CDK4/6 inhibition can control disease without chemotherapy
PATRICIA (SOLTI-1303) [45]	II	MBC (later-line, treatment-refractory)	HR+/HER2+	Trastuzumab	Palbociclib + trastuzumab	Biomarker selection	Identified PAM50 luminal subtype as preferentially benefiting from CDK4/6-based strategies
TOUCH (IBCSG 55-17) [46]	II	Neoadjuvant (chemo-free strategy)	HR+/HER2+	—	Palbociclib + ET + trastuzumab + pertuzumab	Chemo-free feasibility	Demonstrated biological activity of chemotherapy-free dual HER2 + CDK4/6 blockade
DETECT V [16]	II	MBC (1L, chemotherapy-free strategy)	HR+/HER2+	Trastuzumab-based	Ribociclib + anti-HER2 therapy	Translational optimization	Supports CDK4/6 inhibition as antibody partner and explores biomarker-driven escalation

MBC, metastatic breast cancer; HER2+, human epidermal growth factor receptor 2–positive; HR+, hormone receptor–positive; ET, endocrine therapy; 1L, first-line; CDK4/6, cyclin-dependent kinase 4/6.

## Data Availability

No new data were generated or analyzed in this study. Data sharing is not applicable to this article.

## Abbreviations

ADCC	Antibody-Dependent Cellular Cytotoxicity
ADCP	Antibody-Dependent Cellular Phagocytosis
ADC	Antibody–Drug Conjugate
AE	Adverse Event
AKT	Protein Kinase B
CDK4/6	Cyclin-Dependent Kinase 4 and 6
CI	Confidence Interval
ER	Estrogen Receptor
G	Grade
HER2	Human Epidermal Growth Factor Receptor 2
HER2+	Human Epidermal Growth Factor Receptor 2-Positive
HR	Hazard Ratio/Hormone Receptor (depending on context)
HR+/HR−	Hormone Receptor-Positive/Negative
IHC	Immunohistochemistry
MBC	Metastatic Breast Cancer
OS	Overall Survival
pCR	Pathological Complete Response
PFS	Progression-Free Survival
PI3K	Phosphatidylinositol 3-Kinase
TKI	Tyrosine Kinase Inhibitor
